# Management of children with febrile seizures: a Greek nationwide survey

**DOI:** 10.1007/s00431-023-05004-1

**Published:** 2023-05-09

**Authors:** Ioannis Kopsidas, Foteini Eleni Dasoula, Eleni Kourkouni, Adamantia Krepi, Harry Α. Mystakelis, Nikos Spyridis, George Vartzelis

**Affiliations:** 1 Second Department of Pediatrics, School of Medicine, National and Kapodistrian University of Athens, Athens, Greece; 2 Center for Clinical Epidemiology and Outcomes Research, Athens, Greece

**Keywords:** Febrile seizures, Management, Pediatricians, Fever phobia

## Abstract

The purpose of this study was to investigate knowledge, principles, and practices concerning the management of children with febrile seizures among pediatricians in Greece. A cross-sectional study was performed across Greece. Pediatricians completed an anonymous and voluntary 11-item questionnaire about their knowledge, attitudes, and practices with respect to the management of febrile seizures; the survey also collected demographic data. It was first administered in paper form in October 2017. This was followed by an online survey performed between June and August of 2018 and publicized by medical boards across Greece. Descriptive statistics and comparisons between groups were conducted with the significance level set at *p* ≤ 0.05. We recorded 457 responses. Pediatricians admitted to modifying their advice to the parents of children with febrile seizures by suggesting more “aggressive” fever management at low temperatures or systematically (63%), referral to a specialist after any episode of febrile seizures (63%), or hospitalization in a subsequent episode (67%), even though 72% admitted these practices were of no efficacy. Almost one in three pediatricians (28%) believed aggressive management of fever could delay the onset of febrile seizures; increasing age was associated with this perception. A minority (28%) would make parents aware of febrile seizures before a first episode regardless of family history; 38% would do so in the event of family history.

*Conclusions*: Several pediatricians in Greece use outdated and ineffective practices for the management of febrile seizures, despite the availability of updated evidence-based guidelines. Further training of practitioners is needed to bridge this gap.

**Table:** 

**What is Known:** *•Aggressive management of fever at low temperatures with antipyretics, referral to a neurologist, and hospitalization are not supported by evidence or recent guidelines on childhood febrile seizures.* *•Febrile seizures are especially disturbing to uninformed parents, who may be inclined to pursue aggressive but ineffective treatments as a result.*	
**What is New:** *•Pediatricians in Greece use non-evidence-based practices for the management of febrile seizures, even when they are aware that these practices are not effective.* *•Older age increases the likelihood that a pediatrician will pursue guideline non-compliant practices in Greece. At the same time, physicians with over 20 years of experience are more likely to inform parents in advance about febrile seizures.*

**Supplementary Information:**

The online version contains supplementary material available at 10.1007/s00431-023-05004-1.

## Introduction

Febrile seizures are one of the most common pediatric disorders. They usually occur in children between the ages of 6 and 60 months and are divided into two discrete categories: simple and complex [1]. Most are simple febrile seizures, which are generalized (non-focal) seizures lasting fewer than 15 min that do not recur within 24 h. The incidence of febrile seizures is estimated to be from 2–5% [1–3] to 3–8%, thus affecting a significant percentage of the population.

When children are febrile, parents are often concerned and eager to take action. Fever is one of the most common presenting problems in pediatric emergency departments [4]. Fever phobia [5–8] is based on the misconception that fever, if left untreated, can rise and can lead to physical harm and brain damage [9]. Parents’ anxiety may be enhanced by the temporal association of fever with the occurrence of seizures, as a causative relation may be perceived between the two. This can disrupt a family’s daily life, as parents may take days off work and the children may be subjected to unnecessary and costly medical procedures. Of note, almost one-third of the children with a first febrile seizure will have a recurrence, and some will have more than one.

Even though simple febrile seizures are considered benign events with an excellent prognosis [1], the phobia around them persists [6, 7]. Pediatricians play a significant role in this phenomenon by prolonging outdated habits. For example, while the practice of giving antipyretics at a low temperature (i.e., 37.4 °C) has largely been abandoned as ineffective, pediatricians may continue to prescribe antipyretics without clearly communicating to parents that they are used to make the child more comfortable and not because pediatricians are of the opinion that febrile seizure episodes are harmful [5]. Education and anticipatory guidance for pediatric caregivers are needed to help reduce fear of the unknown and to empower caregivers with knowledge of appropriate practices in the event of a seizure (i.e., laying the child on the floor in a side-lying position to prevent aspiration, noting the nature and duration of symptoms, not placing fingers inside the child’s mouth) [8, 10, 11].

Anecdotally, there are indications that pediatricians in Greece resort to outdated practices for the management of febrile seizures. However, to our knowledge, no studies exist that describe this phenomenon. The aim of this study was to assess the knowledge, attitudes, and practices of Greek pediatricians on the management of children with febrile seizures.

## Methods

This was a cross-sectional study that was conducted with an anonymous and voluntary 11-item questionnaire (supplementary file 1) which was developed by selecting themes from previous publications on the management of febrile seizures [12–14] and from the investigators’ own experiences of common practices. Only certified pediatricians were allowed to take a part, and all other specialties were excluded. It was first administered in paper form, with the aid of an associate who filled in the answers, during a pediatric conference in October of 2017 in Athens. Subsequently, medical boards across Greece were asked to inform their members about the existence of the online survey, with data collection performed between June and August 2018; the number of invitations that were actually sent out was not tracked. Data collected included demographic data and information on knowledge, attitudes, and practices around the management of febrile seizures in children; questions on common folk practices and conceptions were also included. All questions were closed-ended, and the majority were presented as “yes/no.” The survey was first tested for clarity by 5 pediatricians and one pediatric neurologist, and adjustments were made based on their feedback. Taking part in the survey required fewer than 5 min. The study was approved by the Scientific Board of the “P. & A. Kyriakou” Children’s Hospital in Athens, Greece.

### Statistical analysis

A descriptive analysis of the data was carried out, and the results were presented as frequencies and percentages. For comparison between the age groups, the chi-square test of independence or Fisher’s exact test was performed, as appropriate. A *p* < 0.05 was considered statistically significant. Analysis was conducted using SPSS v.20. Design and data collection effort ensured that there were no missing data; online data collection had required fields, and in-person questionnaires were filled in by an associate. If a question permitted only one response and 2 responses were checked, the question was not counted in the calculations.

## Results

We obtained 457 responses from pediatricians in Greece. During the first phase, the response rate was 98%, and we collected 144 responses. During the second phase, 313 responses were gathered online. In 2017, there were 3672 registered pediatricians in Greece, so our survey represented 13% of those.

### Sample characteristics

Among respondents, 27% (124/457) were male, and 48% were practicing in the capital city of Athens; 58% had their own private practice, 37% were practicing in a government-owned hospital, and fewer (6.3%) were practicing in a private hospital (note that multiple answers were allowed). Respondents’ characteristics and years of practice can be seen in Table 1. Even though 72% did not think that more aggressive management of fever (for example, more frequent antipyretic administration) could alter the onset of febrile seizures, 63% admitted that they alter their advice for fever management to parents of children with febrile seizures.

**Table 1:** Respondent characteristics and management of febrile seizure

**Demographics**	**n**	%	
**Practice**	457		
Private practice	263	58%	
Private hospital	29	6.3%	
Public hospital	169	37%	
Public rural hospital	11	2.4%	
Retired	7	1.5%	
**Gender**			
Male	124	27%	
Female	333	73%	
**Years of practice/experience**			
< 5	90	20%	
5–10	107	23%	
11–20	118	26%	
> 20	142	31%	
**Region**			
Athens	220	48%	
Thessaloniki	42	9.2%	
Crete	47	10%	
All other areas	148	32%	
**Management**	**Yes**	**No**	**Maybe or depends**
**Does a child with simple febrile seizures need to be hospitalized?**	58 (13%)	362 (80%)	31 (6.9%)
**Believe aggressive management prevents febrile seizures**	45 (9.8%)	330 (72%)	82 (18%)
**Would give different advice for fever management**	282 (61%)	165 (37%)	10 (2.2%)
**What kind of different advice**	292		
Measure temperature more often	208	71%	
Give antipyretics at a lower temperature	257	88%	
< 37.4 °C	32	13%	
37.5–37.9 °C	172	72%	
38–38.4 °C	34	14%	
> 38.5 °C	2	0.8%	
Give antipyretics at a set time regardless of fever	48	16%	
**What kind of advice do you give to parents for a subsequent episode?**			
Supine position	436	95%	
Suppository antipyretic	247	54%	
Don’t put hand in mouth	422	92%	
It’s ok to put a spoon in mouth	27	5.9%	
Count duration of episode	416	91%	
Place under running water	12	2.6%	
Give a slap or two	7	1.5%	
Suppository diazepam	436	95%	
Bring child to me for assessment	250	55%	
Take child to hospital	306	67%	
**When would you refer to a neurologist?**			
Never for simple febrile seizures	333	73%	
After the 1st episode of a simple febrile seizure	57	13%	
Patients with more than 1 episode of simple febrile seizures	208	46%	
After any episode of complex febrile seizures	402	88%	
After any kind of febrile seizures with a 1st degree relative with epilepsy	290	64%	
If it is the parents’ wish	322	71%	

### Management—antipyretics

Almost one in three pediatricians (28%) answered that they assumed aggressive management of fever (i.e., frequent or early administration of antipyretics) could delay the onset of febrile seizures. Most pediatricians (63%) would give different advice on fever management to parents of children with a history of febrile seizures, compared to the ones without, with more frequent measurement of temperature (71%) and administration of antipyretics at a lower temperature (88%); the vast majority (85%) recommended administration at temperatures of or lower than 37.9 °C. A smaller proportion would ask parents to provide antipyretics systematically at set time intervals. Of 330 respondents who did not believe that aggressive management had an effect, almost half (161, 49%) would nonetheless give different advice on the management of temperature to the parents of children with febrile seizures; 79 (49%) were from Athens or Thessaloniki, and almost two out of three (105, 65%) were working in a private environment.

### Management—referral to a neurologist

After a single episode of simple febrile seizures, 13% of pediatricians would refer the child to a neurologist; 46% would do so after the 2nd episode, and 88% would do so after a complex episode. In the setting of a family history of epilepsy, 64% of the pediatricians would make a referral to a child neurologist after any episode of febrile seizure, regardless of type. The parents’ wish for their child to be examined by a specialist was enough reason for 71% of pediatricians to make a referral to a neurologist.

### Management—advice given to parents for a recurrent episode

One in five pediatricians (20%) believed that a following episode of simple febrile seizures, the child should be hospitalized. Of these pediatricians, 13% believed this unconditionally, and 6.9% provided specific conditions. Two out of three pediatricians (67%) advised parents to go to the hospital in the event of another episode of febrile seizures.

Pediatricians were asked to identify, from a set of 10 given sentences, the advice they usually give to parents for the management of a following episode of febrile seizures (Table 1). More than half (54%) would suggest a suppository antipyretic. From 5 to 10% of pediatricians stated they would not give advice to put the child in a recovery position, to avoid putting hands in the child’s mouth, or to record the duration of the episode. However, 27 (5.9%) would advise parents that “it is ok to put a spoon in the child’s mouth,” 12 (3%) that they should put the child under running water, and 7 (21.5%) that it is ok to slap the child.

Prior to a first episode, 38% of pediatricians would inform the parents of the existence of febrile seizures if there was a family history; 34% would inform the parents only after the 1st episode. Only 28% would inform all parents before a first episode, regardless of family history.

### The effect of years of experience

We looked at answers stratified by years of experience and found significant correlations. We found a statistically significant correlation between years of experience and the perception that “aggressive” temperature management can alter the onset of febrile seizures. Pediatricians with experience over 20 years were the ones who mostly answered “yes” or “maybe” to this belief (44%) compared to those with under 5 (18%), 5–10 (14%), or 11–20 years of experience (29%) (*p* = 0.001). Likewise, the percentage of pediatricians who would give different advice for fever management to the parents of children with febrile seizures increased as the years of experience increased (*p* = 0.001), from 42% (less than 5 years of experience) to 77% (more than 20 years of experience). Finally, the respondents with experience over 20 years were the ones who would most frequently inform all parents in advance about the existence of febrile seizures, prior to a first episode or regardless of a history (37%) vs under 5 (24%), 5–10 (26%), and 11–20 years of experience (23%) (*p* = 0.033).

## Discussion

Among pediatricians who care for children with simple febrile seizures in Greece, the topics where knowledge and practice diverged were related to aggressive management of fever with the administration of antipyretics at low temperatures or even systematically, advice for seeking clinical examination at a hospital on a second occurrence, and preemptively informing parents on the existence of febrile seizures. Pediatricians are still giving such advice to parents, contrary to new guidelines [1, 13, 15] and guidance [10, 11, 16, 17] that do not endorse such practices, even though they confess to understanding that such measures are not effective. This could be due to misconceptions about what parents expect from them, as previously described in cases of pediatricians prescribing antibiotics [18, 19].

Febrile seizures are one of the most common pediatric disorders. However, only a fraction (28%) of pediatricians inform all parents in advance about the existence of this issue and its management. Our findings are in concordance with a study on the perceptions and knowledge of parents, where about 2 in 3 parents (62%) were unaware of the existence of febrile seizures before the first event [6]. Almost half of the parents in a study in Turkey did not know what to do during a febrile convulsion [20]. It comes as no surprise that uninformed parents are filled with horror in the event of a febrile seizure and feel helpless to aid their children.

### ﻿Management—antipyretics

It is now known that the increased temperature may be an epiphenomenon related to other processes (immunological, etc.) that may be responsible for the febrile seizures and that antipyretics are ineffective in preventing the temperature from rising during episodes which lead to febrile seizures [21–24]. Even though one recent Japanese trial has shown a prophylactic effect of rectal acetaminophen every 6 h for 24 h on the short-term recurrence of seizures [25], a recent Cochrane systematic review concludes that there was no benefit in the use of acetaminophen, ibuprofen, or diclofenac in the management of febrile seizures [26]. Prior misconceptions that antipyretics can prevent febrile seizures or febrile seizures and brain damage [27–29] can be perpetuated by physicians giving advice that may imply that antipyretics can have an effect on the emergence of fever and seizures. The misconception on the role of antipyretics has been described before in the literature among healthcare workers, and in some cases, aggressive management included alternating antipyretics [29] and could perpetuate parents’ misconceptions as well [6]. The relationship developed between pediatricians and caregivers can either be positive and make the caregivers confident in the way they manage their child’s fever, or, in the absence of trust, it can lead to fever phobia and fever overtreatment [7]. Additionally, evidence suggests that in children, rectal diazepam and midazolam (either intranasal, buccal, or IM) are probably effective at stopping seizures that last at least 5 min [30].

### Management—hospital referral

When managing convulsing children, bacterial meningitis is the major concern of a physician. However, the risk of bacterial meningitis in children 6 to 11 or 18 months of age with a first simple febrile seizure, without any other signs or symptoms, is very low [31, 32]. Since simple febrile seizures in children are not associated with an increased likelihood of urinary tract infections, pneumonia, bacteremia, or bacterial meningitis [33, 34], well-appearing children with simple febrile seizures do not need routine diagnostic testing, including laboratory exams, neuroimaging, or electroencephalography, unless required to determine the underlying cause of the fever [34].

Therefore, advising the parents to visit the hospital for a case of simple febrile seizures, in the absence of a concern for the presence of meningitis or intracranial infection or with another identified source for the fever, can lead to unnecessary hospitalization and further invasive diagnostic procedures (i.e., lumbar puncture, blood cultures, electroencephalograms, neuroimaging) and add an unnecessary burden to both families and the healthcare system. In our study, 13% of pediatricians surveyed would make a referral to a neurologist after a single case of a simple febrile seizure, and 67% would advise that the child be taken to the hospital on a subsequent episode.

The implementation of the 2011 AAP guidelines lead to a decrease in diagnostic procedures, hospital admissions, and total mean costs from $1523 in 2005 to $605 in 2019 [33]. Similarly, the introduction of the new guidelines in 2015 in Japan was followed by a reduction in lumbar punctures and blood examinations ordered [15]. Overall, there seems to be a paucity of data on the treatment costs of children with febrile seizure events, and they are mostly dependent on the clinical workup [6]. Even though there are no data for the burden of simple febrile seizures to the healthcare system in Greece, one can expect that adhering to best practices will lead to subsequent gains for the healthcare system as well as for parents, as they would not need to take days off to be at the hospital.

### Not a unique problem

The literature shows that there are gaps in what the caregivers of children know and in the knowledge and practices of pediatricians and pediatric healthcare providers. Different aspects of the same problem have been reported in Korea [35], Iran [36], Turkey, the USA [37], Saudi Arabia [38], Iraq [39], India [40], Germany [6], and Japan [15, 41] among other countries. The reports span across at least the last 15 years, showing that it an ongoing problem that can affect different populations at different times. The common denominator is that there is still work to be done in the matter of increasing awareness and knowledge and implementing best practices in the management of children with febrile convulsions.

### The effect of age

In the paper by Sakai et al. [41], pediatricians with experience over 20 years had a better understanding and management of febrile seizures. In our study, in contrast, it seems that increasing age is a factor for guideline non-compliant practices. At the same time, physicians with over 20 years of experience are more likely to inform all parents in advance about febrile seizures.

### Training

Pediatricians give information to parents on diet, nutrition, immunization, and techniques to avoid accidents. Well-planned educational initiatives on fever and febrile seizure management can lead to improved parental knowledge, less fear, and better practices in the management of fever [5] or febrile seizures [36]. In order to educate parents, however, one needs to educate the educator—in this case, the pediatrician—as our study showed. In this direction, the development of guidelines may lead to better practices [15]. Consensus statements [8] can be helpful in creating training programs for healthcare workers and providing parents with solid information and recommendations. Currently, in Greece, there is no requirement for re-certification or keeping up to date through training that offers CME or other accreditation, which can be problematic, especially for the pediatricians who are not affiliated or working with a hospital. The issuing of new guidelines can be tracked better when working within hospitals [15]. Training could be offered at pediatric seminars or even through the medical associations to which they are obliged to renew their membership yearly.

### Limitations

This study has certain limitations that arise from the way it was performed. The survey was first given in paper form with a 98% response rate, and it was then disseminated to pediatricians by email through their medical associations in many, but not all geographic regions of Greece. Not all associations responded to our call, and from the ones that did respond, we cannot account for emails sent successfully without bouncing for any reason or how many emails were actually read. Therefore, our results may not be generalizable since not all pediatricians were contacted, a survey response rate could not be calculated, and the survey was biased toward physicians who were comfortable with email and an online survey. At the same time, this may suggest that the problem we describe has been underestimated, as the doctors who took part in the survey are those who attend seminars and respond to emails—i.e., those who make use of the internet—and thus are probably highly motivated and educated pediatricians. However, it should be noted that in 2017, there were 3672 registered pediatricians throughout Greece [42], and we managed to receive responses from only 457, or 13%. Other information that could help us estimate if our sample was representative (of, for example, age, sex, and geographical area) was not available to us, and even though we contacted the Panhellenic Medical Association, we received no answer. Even though 73% of the respondents stated that they would never refer a patient to a neurologist for simple seizures, 46% responded they would refer after 2 or more episodes of simple febrile seizures. We cannot explain this discrepancy, especially since these questions were yes/no questions and not multiple choice. Even though our data are only from Greece, it is not clear that they can be generalized to other countries, in Europe or elsewhere, especially in view of different healthcare systems, training programs, and socioeconomic variables that may affect pediatricians’ level of education. However, the literature shows that this is an international and ongoing matter. Raising awareness could be the first step in identifying more countries with similar problems and ultimately bridging the gap between theory and practice.

Despite these limitations, our study provides ample evidence that there is a gap between the knowledge and practices of pediatricians in Greece in their management of febrile seizures.

## Conclusion

Even though new guidelines for simple febrile seizures do not endorse aggressive management of fever with antipyretics at low temperatures, referral to a neurologist, or hospitalization, pediatricians in Greece continue to use such practices, even though in some cases they do not believe in their efficacy. This can have increase the anxiety felt by parents and the burden imposed on the child and the healthcare system. Training initiatives could help to tackle this problem.

## Supplementary Information

Below is the link to the electronic supplementary material.

Supplementary file1 (PDF 467 kb)

## Data Availability

All data generated or analyzed during this study are included in this article.

## Code availability

N/A.

## References

[1] Subcommittee on Febrile Seizures (2011). Febrile seizures: guideline for the neurodiagnostic evaluation of the child with a simple febrile seizure. Pediatrics.

[2] Jones T, Jacobsen SJ (2007). Childhood febrile seizures: overview and implications. Int J Med Sci.

[3] Gordon KE, Dooley JM, Camfield PR (2001). Treatment of febrile seizures: the influence of treatment efficacy and side-effect profile on value to parents. Pediatrics.

[4] Armon K, Stephenson T, Gabriel V (2001). Determining the common medical problems to an accident and emergency department. Arch Dis Child.

[5] May A, Bauchner H (1992). Fever phobia : the pediatrician’s. Pediatr Rev Am Acad Pediatr.

[6] Rice SA, Müller RM, Jeschke S (2022). Febrile seizures: perceptions and knowledge of parents of affected and unaffected children. Eur J Pediatr.

[7] Merlo F, Falvo I, Caiata-Zufferey M (2023). New insights into fever phobia: a pilot qualitative study with caregivers and their healthcare providers. Eur J Pediatr.

[8] Loussouarn A, Devlin A, Bast T (2021). Consensus statements on the information to deliver after a febrile seizure. Eur J Pediatr.

[9] Walsh A, Edwards H (2006). Management of childhood fever by parents: literature review. J Adv Nurs.

[10] Ma L, McCauley SO (2018). Management of pediatric febrile seizures. J Nurse Pract.

[11] Warden CR, Zibulewsky J, Mace S (2003). Evaluation and management of febrile seizures in the out-of-hospital and emergency department settings. Ann Emerg Med.

[12] Natsume J, Hamano S, Iyoda K (2017). New guidelines for management of febrile seizures in Japan. Brain Dev.

[13] Committee S, on Quality Improvement and Management S on FS,  (2008). Febrile seizures: clinical practice guideline for the long-term management of the child with simple febrile seizures. Pediatrics.

[14] Desnous B, Goujon E, Bellavoine V (2011). Perceptions of fever and fever management practices in parents of children with Dravet syndrome. Epilepsy Behav.

[15] Tanaka M, Natsume J, Hamano S-I (2020). The effect of the guidelines for management of febrile seizures 2015 on clinical practices: nationwide survey in Japan. Brain Dev.

[16] Fetveit A (2008). Assessment of febrile seizures in children. Eur J Pediatr.

[17] Laino D, Mencaroni E, Esposito S (2018). Management of pediatric febrile seizures. Int J Environ Res Public Health.

[18] Mangione-Smith R, McGlynn EA, Elliott MN (2001). Parent expectations for antibiotics, physician-parent communication, and satisfaction. Arch Pediatr Adolesc Med.

[19] Mangione-Smith R, McGlynn EA, Elliott MN (1999). The relationship between perceived parental expectations and pediatrician antimicrobial prescribing behavior. Pediatrics.

[20] Kayserili E, Ünalp A, Apa H (2008). Parental knowledge and practices regarding febrile convulsions in Turkish children. Turk J Med Sci.

[21] van Stuijvenberg M, Derksen-Lubsen G, Steyerberg EW (1998). Randomized, controlled trial of ibuprofen syrup administered during febrile illnesses to prevent febrile seizure recurrences. Pediatrics.

[22] Rosenbloom E, Finkelstein Y, Adams-Webber T, Kozer E (2013). Do antipyretics prevent the recurrence of febrile seizures in children? A systematic review of randomized controlled trials and meta-analysis. Eur J Paediatr Neurol.

[23] Strengell T, Uhari M, Tarkka R (2009). Antipyretic agents for preventing recurrences of febrile seizures: randomized controlled trial. Arch Pediatr Adolesc Med.

[24] Richardson M, Purssell E (2015). Who’s afraid of fever?. Arch Dis Child.

[25] Murata S, Okasora K, Tanabe T (2018). Acetaminophen and febrile seizure recurrences during the same fever episode. Pediatrics.

[26] Offringa M, Newton R, Cozijnsen MA, Nevitt SJ (2017). Prophylactic drug management for febrile seizures in children. Cochrane Database Syst Rev.

[27] Sarrell M, Cohen HA, Kahan E (2002). Physicians’, nurses’, and parents’ attitudes to and knowledge about fever in early childhood. Patient Educ Couns.

[28] Kolahi A-A, Tahmooreszadeh S (2009). First febrile convulsions: inquiry about the knowledge, attitudes and concerns of the patients’ mothers. Eur J Pediatr.

[29] Martins M, Abecasis F (2016). Healthcare professionals approach paediatric fever in significantly different ways and fever phobia is not just limited to parents. Acta Paediatr.

[30] Glauser T, Shinnar S, Gloss D (2016). Evidence-based guideline: treatment of convulsive status epilepticus in children and adults: report of the guideline committee of the American epilepsy society. Epilepsy Curr.

[31] Guedj R, Chappuy H, Titomanlio L (2015). Risk of bacterial meningitis in children 6 to 11 months of age with a first simple febrile seizure: a retrospective, cross-sectional, observational study. Acad Emerg Med.

[32] Kimia AA, Capraro AJ, Hummel D (2009). Utility of lumbar puncture for first simple febrile seizure among children 6 to 18 months of age. Pediatrics.

[33] Raghavan VR, Porter JJ, Neuman MI, Lyons TW (2021). Trends in management of simple febrile seizures at US children’s hospitals. Pediatrics.

[34] Smith DK, Sadler KP, Benedum M (2019). Febrile seizures: risks, evaluation, and prognosis. Am Fam Physician.

[35] Oh W-O, Heo YJ, Suk MH, Lee A (2021). Korean childcare providers’ knowledge, attitudes, concerns, and practices of febrile convulsions. Int J Environ Res Public Health.

[36] Sharifirad G, Najimi A, Dolatabadi N, Esmaeili A (2013). The effect of educational program on knowledge, attitude and practice of mothers regarding prevention of febrile seizure in children. J Educ Health Promot.

[37] Carapetian S, Hageman J, Lyons E (2015). Emergency department evaluation and management of children with simple febrile seizures. Clin Pediatr (Phila).

[38] Bashiri FA, Al Shalawi AA, Hamad MH et al (2018) Assessment of physicians’ knowledge and attitudes in the management of febrile seizures. Neurosciences 23:314–319. 10.17712/nsj.2018.4.20180097 10.17712/nsj.2018.4.20180097 PMC8015563 30351289

[39] Shibeeb N, Altufaily YS (2019). Parental knowledge and practice regarding febrile seizure in their children. Med J Babylon.

[40] Srinivasa S, Anjum SK, Patel S et al (2018) Parental knowledge, attitude and practices regarding febrile convulsion. Int J Contemp Pediatr 5:515–519. 10.18203/2349-3291.ijcp20180546

[41] Sakai R, Okumura A, Shimizu T, Marui E (2009). Current explanations regarding febrile seizures provided by pediatricians in Tokyo. Dev Med Child Neurol.

[42] Hellenic Statistical Authority {ELSTAT} - Physicians and dentists 2017. https://www.statistics.gr/el/statistics/-/publication/SHE09/2017. Accessed 21 May 2022

