# Supplementary material for: Management of children with febrile seizures: a Greek nationwide survey
Source: Eur J Pediatr. 2023 May 9;182(7):3293–300. doi: 10.1007/s00431-023-05004-1 (PMC10353953; doi:10.1007/s00431-023-05004-1)
Supplement: Supplementary file 1 — Supplementary file1 (PDF 467 kb) [file 431_2023_5004_MOESM1_ESM.pdf]

ΕΘΝΙΚΟ ΚΑΙ ΚΑΠΟΔΙΣΤΡΙΑΚΟ
ΠΑΝΕΠΙΣΤΗΜΙΟ ΑΘΗΝΩΝ
Β' Παιδιατρική Κλινική
Διευθύντρια : Καθηγήτρια Μ.Τσολιά

SURVEY ON THE MANAGEMENT OF FEBRILE SEIZURES BY PEDIATRICIANS IN GREECE

The 2nd Department of Pediatrics of the National and Kapodistrian University of Athens is conducting a voluntary and anonymous survey on the management of febrile seizures by pediatricians in Greece. This questionnaire should not take more than 3-5 minutes to complete.

A. Demographics

1. **Sex**
   - ☐ Male
   - ☐ Female

2. Where do you practice pediatrics (you can choose more than one)?
- ☐ Private Practice
- ☐ Private Hospital
- ☐ Public Hospital
- ☐ Public Primary healthcare structure / Public Rural Hospital (primary care)
- ☐ Retired / Not currently working

3. How many years have you been practicing pediatrics (Including residency) ____
(if less than a year please enter 0)

4. Region of your practice: ________________________

B. Febrile seizures Management

1. In the case of a child with known history of febrile seizures do you believe that 'aggressive' management of fever (e.g. regular administration of antipyretics) **prevents febrile seizures?**
- ☐ Yes
- ☐ No
- ☐ Maybe

2. Do you change in any way the advice that you give for fever management to parents of children with known history of febrile seizures, compared to the ones with no history of febrile seizures?
- ☐ Yes
- ☐ No
- ☐ Other (it depends) – Διευκρινίστε: ___________________________

3. If you answered Yes/Other, in which way do you alter the advice:

Measure temperature more often	☐ Yes ☐ No
Give antipyretics at a lower temperature	☐ Yes ☐ No
If yes, then at which temperature: ____ °C	
Administer antipyretics regularly (every X hours regardless of temperature)	☐Yes ☐ No

4. What Kind of advice do you give to parents of a subsequent episode of febrile seizure?

«Put the child in supine position»	☐Yes ☐No
«Give suppository antipyretic»	☐Yes ☐No
«Don't put your hand in the child's mouth»	☐Yes ☐No
«If you want, you can put a spoon in the child's mouth»	☐Yes ☐No
«Measure the duration of the episode»	☐Yes ☐No
«Place the child under running water»	☐Yes ☐No
«Give a facial stimulation»	☐Yes ☐No
«If after 3-5 minutes seizures continue administer suppository diazepam»	☐Yes ☐No
«Bring child to me for assessment»	☐Yes ☐No
«Go to the hospital»	☐Yes ☐No

5. After an episode of febrile seizure, when would you refer to a neurologist?

Never for simple febrile seizures	☐Yes ☐No
After the 1st episode of simple febrile seizure	☐Yes ☐No
To patients with more than 1 episode of simple febrile seizure	☐Yes ☐No
After any episode of complex febrile seizures	☐Yes ☐No
After any kind of febrile seizures if there is 1st degree relative with epilepsy	☐Yes ☐No
If it is the parents' wish	☐Yes ☐No

6. Do you believe that hospitalization is needed in every consequent episode of simple febrile seizures?

- ☐ Yes
- ☐ No
- ☐ It depends / Other. Explain: ________________________

7. When do you inform parents on the existence of Febrile seizures?

- ☐ Only after the 1st episode
- ☐ In advance, all parents
- ☐ In advance only if there is family history

**Comments**

Thank you for your time
